# Recent influenza activity in tropical Puerto Rico has become synchronized with mainland US

**DOI:** 10.1111/irv.12744

**Published:** 2020-07-02

**Authors:** Gabriela Paz–Bailey, Talia M. Quandelacy, Laura E. Adams, Sonja J. Olsen, Lenee Blanton, Jorge L. Munoz-Jordan, Matthew Lozier, Luisa I. Alvarado, Michael A. Johansson

**Affiliations:** ^1^ Centers for Disease Control and Prevention San Juan Puerto Rico; ^2^ Centers for Disease Control and Prevention Atlanta Georgia; ^3^ Saint Luke’s Episcopal Hospital and Ponce Health Sciences University Ponce Puerto Rico

**Keywords:** COVID-19, influenza, Puerto Rico, synchrony, trends, United States

## Abstract

**Background:**

We used data from the Sentinel Enhanced Dengue Surveillance System (SEDSS) to describe influenza trends in southern Puerto Rico during 2012‐2018 and compare them to trends in the United States.

**Methods:**

Patients with fever onset ≤ 7 days presenting were enrolled. Nasal/oropharyngeal swabs were tested for influenza A and B viruses by PCR. Virologic data were obtained from the US World Health Organization (WHO) Collaborating Laboratories System and the National Respiratory and Enteric Virus Surveillance System (NREVSS). We compared influenza A and B infections identified from SEDSS and WHO/NREVSS laboratories reported by US Department of Health and Human Services (HHS) region using time series decomposition methods, and analysed coherence of climate and influenza trends by region.

**Results:**

Among 23,124 participants, 9% were positive for influenza A and 5% for influenza B. Influenza A and B viruses were identified year‐round, with no clear seasonal patterns from 2012 to 2015 and peaks in December‐January in 2016‐2017 and 2017‐2018 seasons. Influenza seasons in HHS regions were relatively synchronized in recent years with the seasons in Puerto Rico. We observed high coherence between absolute humidity and influenza A and B virus in HHS regions. In Puerto Rico, coherence was much lower in the early years but increased to similar levels to HHS regions by 2017‐2018.

**Conclusions:**

Influenza seasons in Puerto Rico have recently become synchronized with seasons in US HHS regions. Current US recommendations are for everyone 6 months and older to receive influenza vaccination by the end of October seem appropriate for Puerto Rico.

## 1 BACKGROUND

Influenza is a significant public health problem globally. Seasonal influenza has a high disease burden, reducing school attendance, increasing worker absenteeism and impacting daily productivity.^1^ Since 2010, CDC estimates that influenza is associated with between 9.3‐49 million illnesses, 140 000‐960 000 hospitalizations and 12 000‐79 000 deaths each year in the United States (US).^2^ Though antivirals treat infection, vaccination remains the primary tool to prevent influenza‐associated morbidity and mortality.^3^ The US Advisory Committee on Immunization Practices (ACIP) recommends routine annual influenza vaccination for individuals, aged ≥6‐months who do not have contraindications.^4^ Influenza immunization programmes must consider disease seasonality to most effectively use immunization.^5^ As a US Territory, Puerto Rico follows the same seasonal influenza vaccination recommendations as the continental US. However, given its tropical climate and distant geographic location, Puerto Rico's seasonality may differ compared with the continental United States. This difference may impact public health measures, for example when to recommend the annual influenza vaccine in Puerto Rico.

In high‐income temperate countries, influenza has been well described. Most seasonal epidemics in temperate regions occur during the winter months, between November and March in the Northern Hemisphere and between April and September in the Southern Hemisphere.^6^ These seasonal patterns are thought to be driven by annual changes in climate, contact rates, human immunity and other factors.^1^, ^7^, ^8^ Some tropical and subtropical regions experience annual epidemics coinciding with local rainy seasons,^9^ whereas others have semi‐annual epidemics or year‐round influenza activity without well‐defined influenza seasons.^10^, ^11^ Influenza seasonality in tropical and subtropical regions is less understood.^9^

Few studies examined influenza in Puerto Rico, and none examine Puerto Rico influenza trends in relation to patterns in the continental United States. Comparing seasonal patterns of influenza in Puerto Rico to seasonality in the continental United States would help inform immunization programmes, surveillance and mitigation measures on the island. We analysed data from enhanced acute febrile illness (AFI) surveillance sites in southern Puerto Rico during 2012 to 2018 to define typical periods of influenza activity. To better understand the temporal dynamics of influenza epidemics and possible drivers, we used time series decomposition methods to isolate seasonal oscillations of influenza epidemics in Puerto Rico and to compare them to influenza epidemics in US Health and Human Service [HHS] regions (Figure S1). We also explored coherence between climate and influenza trends for Puerto Rico and HHS regions, to determine whether climatic differences explained differences in influenza trends.

## 2 METHODS

### 2.1 Sentinel enhanced dengue surveillance system

The Sentinel Enhanced Dengue Surveillance System (SEDSS)^12^ recruits febrile patients presenting at emergency departments (ED) and one outpatient clinic in Ponce, Puerto Rico. SEDSS started in May 2012 at Saint Luke's Episcopal Hospital, a 427 inpatient bed tertiary care hospital that receives ~60 000 patients/year, and is the regional paediatric referral hospital for the Ponce and Mayaguez (~500 000 residents) Health Districts. The Outpatient Acute Care Clinic (2016‐present) services ~20 000 patients/year. Guayama Menonita Hospital (2013‐2015) is a 161‐bed secondary care hospital site that receives ~35 000 patients per year from southeastern Puerto Rico (~50 000 residents), and participated in SEDSS from 2013‐2015. The municipality of Ponce has a population of 133 191 persons for 2018. The age distribution is 5% are ≤5 years, 20% 5‐18 years, 22% ≥65 years (https://www.census.gov/).

Patients presenting to the SEDSS sites were queried for current fever (body temperature of ≥38.0°C [oral] or ≥38.5°C [axillary]), or having reported a history of fever within the last 7 days. Those responding positively for fever were enrolled. Blood, urine, and combined nasopharyngeal and oropharyngeal swabs were collected from enrolled patients, and diagnostic testing was performed. Upper respiratory tract specimens were tested for influenza A and B viruses, adenovirus, respiratory syncytial virus (RSV), coronavirus, parainfluenza viruses and human metapneumovirus by real‐time reverse transcription polymerase chain reaction (RT–PCR) as previously described.^13^, ^14^, ^15^ For each respiratory pathogen, a positive result was defined as nucleic acid detected by PCR (cycle threshold cut–off 37.0). SEDSS participants provided demographic, history of exposure and clinical data during enrolment interview.

All enrolled patients provided informed consent. The Ponce Medical School Foundation and the US Centers for Disease Control and Prevention (CDC) ethics committees reviewed and approved the SEDSS study protocol.

### 2.2 US influenza viral surveillance

Virologic surveillance data from the 40th week of 2012 to the end of 2018 were obtained from the US World Health Organization (WHO) Collaborating Laboratories and the National Respiratory and Enteric Virus Surveillance System (NREVSS). The WHO Collaborating Laboratories System is maintained by CDC’s Influenza Division and the NREVSS is maintained by CDC’s Division of Viral Diseases. The data included the total number of respiratory specimens tested, and the number positive for influenza types A and B viruses each week. Data were collected, analysed and aggregated nationally and by HHS regions (Figure S1).^1^Region 1 includes: Connecticut, Maine, Massachusetts, New Hampshire, Rhode Island, and Vermont. Region 2 includes: New Jersey, New York, Puerto Rico, and the Virgin Islands. Region 3 includes: Delaware, District of Columbia, Maryland, Pennsylvania, Virginia, and West Virginia. Region 4 includes: Alabama, Florida, Georgia, Kentucky, Mississippi, North Carolina, South Carolina, and Tennessee. Region 5 includes: Illinois, Indiana, Michigan, Minnesota, Ohio, and Wisconsin. Region 6 includes: Arkansas, Louisiana, New Mexico, Oklahoma, and Texas. Region 7 includes: Iowa, Kansas, Missouri, and Nebraska. Region 8 includes: Colorado, Montana, North Dakota, South Dakota, Utah, and Wyoming. Region 9 includes: Arizona, California, Hawaii, Nevada, American Samoa, Common wealth of the Northern Mariana Islands, Federated States of Micronesia, Guam, Marshall Islands, and Republic of Palau. Region 10 includes: Alaska, Idaho, Oregon, and Washington.


### 2.3 Data analyses

We compared the number of confirmed influenza A and B cases in SEDSS to virologic surveillance data collected by US WHO and NREVSS laboratories by HHS region. Weekly numbers of influenza cases combined influenza A and B virus types. The reporting period for each influenza seasons begin during week 40 and ends week 39 of the following year. For weekly case time series in Puerto Rico and HHS regions, the peak week was the week with the maximum number of positive cases. To compare the difference in timing (in weeks) of influenza epidemics between SEDSS and HHS regions, we used continuous wavelet transforms to isolate the seasonal component of the time series data. First, case count time series were normalized by logging the weekly case counts and standardizing by the mean and standard deviation of the time series. The seasonal time series for each region was reconstructed using Morlet wavelet decomposition to extract the major seasonal component (period: 40‐56 weeks) from the normalized case count time series. This range allowed us to capture fluctuations around the 52‐week yearly cycle. Then, we calculated coherence and phase difference analyses between the seasonal influenza A and B component from SEDSS to the seasonal influenza component of each HHS region.^16^ Coherence estimated the relatedness of the time series, with a coherence of 1 indicating the time series are exactly identical though possibly lagged, while a coherence of 0 indicates no association between the two time series. Seasonal epidemics in two regions were considered to be in phase (ie, occurring at the same time), if there was no difference between their phases (ie relative timing in weeks). Relative to the reference location, Puerto Rico, a positive phase difference would indicate that seasonal epidemics occurred earlier in Puerto Rico than the matched HHS region. In contrast, a negative phase difference indicated that seasonal epidemics were later in Puerto Rico than in the HHS Region. Results based on peak timing were confirmed by the wavelet phase analysis, which considers the entire epidemic cycle.^17^

To explore if differences in seasonality between Puerto Rico and HHS regions were due to differences in climate, we obtained regional climate data (absolute humidity, temperature and precipitation) from 2012 to 2018 from the North American Regional Reanalysis data set from the National Oceanic and Atmospheric Administration (NOAA).^18^ Daily climate data were temporally and spatially aggregated to weekly means for each HHS region and Puerto Rico. Weekly mean time series data were normalized. In order to analyse the possible relationship to climate, we extended the timeframe considered for the seasonal component to 40‐80 weeks to include potential climatic trends that might occur outside the typical seasonal periodicity. For each region, we used the Morlet wavelet decomposition to extract the major seasonal component (period: 40‐80 weeks) for weekly influenza cases and the weekly mean absolute humidity, temperature and precipitation.

### 2.4 Role of funding source

The US CDC funded the study, and participated in the study design, data analysis, data interpretation and preparation of the manuscript. All authors had full access to study data, and all authors had final responsibility for the decision to submit for publication.

## 3 RESULTS

Of 78 822 febrile patients presenting to SEDSS sites during 2012‐2018, 28 280 were offered participation and 23 124 (82%) participants enrolled in SEDSS. Among participants, 52% were female, and 60% were aged ≤17 years. Half of the participants were from the Ponce municipality, and most (72%) presented within 3 days of illness onset. While 82% were sent home after evaluation and treatment, 16% had hospital admittance. From 2012‐2013, 20% of the total enrollees were recruited, 20% in 2014‐2015, 43% in 2016‐2017 and 17% in 2018. Participants’ clinical and demographic characteristics did not change between years (Table 1). Overall, 14% of participants were positive for influenza; 9% for influenza A and 5% for influenza B viruses. Although there were no differences by gender, the percentage of participants with positive results for influenza A or B viruses differed by age (*P* < .001) and days after symptom onset (*P* < .001; Table 2).

**Table 1:** Characteristics of participants by year of recruitment, Sentinel Enhanced Dengue Surveillance System, Ponce, Puerto Rico, 2012‐2018

Participant Characteristics	Overall		2012‐2013		*2014‐2015*		*2016‐2017*		*2018*	
	N	%	N	%	N	%	N	%	N	%
Gender										
Female	11 924	51.6	2282	49.6	2383	50.0	5264	53.5	1995	51.0
Male	11 200	48.4	2320	50.4	2388	50.1	4578	46.5	1914	49.0
Age										
<1 y	1997	8.6	354	7.7	535	11.2	790	8.0	318	8.1
1‐5 y	6395	27.7	1128	24.5	1582	33.2	2483	25.2	1202	30.8
6‐17 y	5430	23.5	1350	29.3	1141	23.9	2000	20.3	939	24.0
18‐64 y	7874	34.1	1513	32.9	1234	25.9	3923	39.9	1204	30.8
65 + y	1428	6.2	257	5.6	279	5.9	646	6.6	246	6.3
Municipality of residence										
Ponce	11 747	50.8	2021	43.9	2075	43.5	5608	57.0	2043	52.3
Juana Diaz	2215	9.6	407	8.8	413	8.7	1020	10.4	375	9.6
Peñuelas	2076	9.0	161	3.5	219	4.6	1150	11.7	546	14.0
Guayama	1289	5.6	590	12.8	676	14.2	15	0.2	8	0.2
Villalba	1088	4.7	236	5.1	211	4.4	484	4.9	157	4.0
Other municipality	4709	20.4	1187	25.8	1177	24.7	1565	15.9	780	20.0
Days post‐onset of symptoms										
<3 d	16 632	71.9	2911	63.3	3784	79.3	6987	71.0	2950	75.5
3‐7 d	6416	27.8	1655	36.0	986	20.7	2852	29.0	923	23.6
8 + d	76	0.3	36	0.8	1	0.0	3	0.0	36	0.9
Disposition outcome										
Sent home	19 051	82.4	3192	69.4	3724	78.1	8665	88.0	3470	88.8
Admitted	3707	16.0	1378	29.9	1024	21.5	964	9.8	341	8.7
Transferred	171	0.7	14	0.3	14	0.3	109	1.1	34	0.9
Died	28	0.1	12	0.3	4	0.1	10	0.1	2	0.1
Other	167	0.7	6	0.1	5	0.1	94	1.0	62	1.6
Total	23 124	100	4602	100	4771	100	9842	100	3909	100

**Table 2:** Characteristics of participants with Influenza A and Influenza B, Sentinel Enhanced Dengue Surveillance System, Ponce, Puerto Rico, 2012‐2018

Participant Characteristics	Total	All Influenza		Influenza A		Influenza B	
	N	n	%	n	%	n	%
Gender							
Female	11 924	1619	13.6	1051	8.8	568	4.8
Male	11 200	1595	14.2	985	8.8	610	5.5
Age							
<1 y	1997	128	6.4	91	4.6	37	1.9
1‐5 y	6395	680	10.6	468	7.3	212	3.3
6‐17 y	5430	1031	19.0	517	9.5	514	9.5
18‐64 y	7874	1194	15.2	846	10.7	348	4.4
65 + y	1428	181	12.7	114	8.0	67	4.7
Days post‐onset of symptoms							
<3 d	16 632	2427	14.6	1635	9.8	792	4.8
3‐7 d	6416	778	12.1	394	6.1	384	6.0
8 + d	76	9	11.8	7	9.2	2	2.6
Disposition outcome							
Sent home	19 051	2864	15.0	1800	9.5	1064	5.6
Admitted	3707	320	8.6	217	5.9	103	2.8
Transferred	171	9	5.3	6	3.5	3	1.8
Died	28	3	10.7	3	10.7	.	.
Other	167	18	10.8	10	6.0	8	4.8
Total	23 124	3214	13.9	2036	8.8	1178	5.1

**Figure 1:**
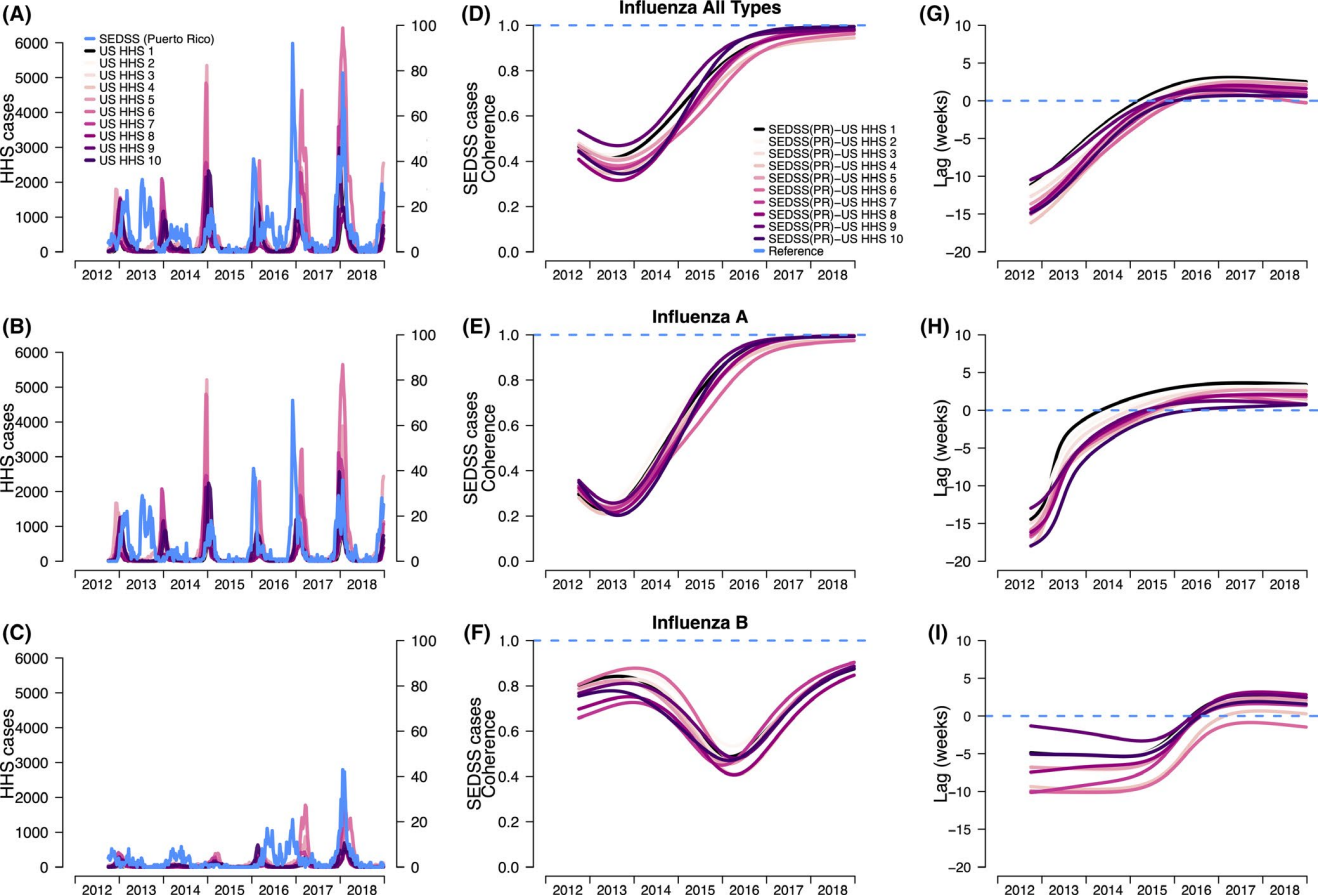
A, Number of influenza cases overall (1A‐C) and by type in the Sentinel Enhanced Dengue Surveillance System (SEDSS), Southern Puerto Rico (PR) and US HHS regions (US WHO/NRVESS), (B). Coherence (1D‐F) comparing the similarity between the influenza patterns in the US HHS regions with SEDSS (PR), and (C). Phase differences (1G‐I) comparing the timing of SEDSS (PR) influenza seasonal epidemics to those in each of the US HHS regions. Seasonal decomposition was defined as a 40‐ to 56‐wk period. Colour lines refer to the comparisons of the weekly seasonal time series of SEDSS (PR) to each US HHS region (PR‐HHS). For coherence, the dashed line at 1 refers to identical seasonal patterns of the US HHS region and the SEDSS (PR), whereas 0 refers to no association of the two time series. For phase differences, negative values indicate the seasonal pattern of SEDSS (PR) influenza cases is behind US HHS regions, and positive values indicate the seasonal pattern of SEDSS (PR) cases is ahead of US HHS regions

We compared the time series and peak timing of overall influenza and type‐specific patterns in Puerto Rico to the HHS regions. In Puerto Rico (SEDSS), influenza peaks varied substantially between 2013 and 2018 (Figure 1A). Specifically, influenza A virus had an atypical summer peak in 2013, but had subsequent peaks in the winter months (December, January and February) with similar timing to those in the HHS regions (Figure 1B). In the HHS regions, influenza peaked consistently during the winter months (December and January) throughout the study period. For influenza B virus, long‐term patterns in SEDSS were less clear with some peaks in the summer (2016) and others in the winter months (2017‐2018). Only 2017‐2018 appeared to be closely aligned with HHS regions, where influenza B virus peaked in the winter and early spring (January to March; Figure 1C). The seasonal components from the time series confirmed these trends (Figure S2A‐C).

We performed coherence and phase analyses to estimate the relatedness and relative timing (in weeks) of the influenza time series in SEDSS, Puerto Rico to those in each of the US HHS regions. Comparing SEDSS to the HHS regions for overall influenza, there was low coherence during 2013‐2015, and high coherence in 2016 (Figure 1D). Similarly, influenza A virus in SEDSS had high coherence with influenza A virus in each HHS time series in later years (Figure 1E). Influenza B virus coherence differed from A. Influenza B virus coherence was higher in the early years, decreasing to roughly 0.40‐0.45 in 2015‐2016, then increased in 2016‐2017 (Figure 1F). The average annual coherence of influenza was highest when comparing US HHS Region 2 (0.66) and US HHS Region 9 (0.67). It should be noted that US HHS region 2 includes Puerto Rico (along with the US Virgin Island, New York, and New Jersey). The southern states are represented in US HHS region 4 (Alabama, Florida, Georgia, Kentucky, Mississippi, North Carolina, South Carolina, and Tennessee), but this region had lower coherence with Puerto Rico (0.52), suggesting they do not have more similar influenza patterns (Table S1). Similar coherence patterns are also observed when looking at influenza A and B.

From the phase analyses, the seasonal components of the overall time series showed a synchronization between SEDSS and the HHS regions across the study period (Figure 1G‐I). From 2013 to 2015, the SEDSS seasonal influenza patterns lagged behind the HHS regions by as much as 12‐18 weeks. However, this pattern inverted in 2016, after which the seasonal patterns in SEDSS led the patterns in the HHS regions by 0‐5 weeks. Influenza A and B viruses showed distinct patterns when comparing SEDSS to HHS regions. The sporadic and off‐season dynamics of influenza A virus in SEDSS in 2013‐2014 led to a high degree of asynchrony with the HHS regions. However, those dynamics quickly changed beginning in 2014, with seasonal influenza A having similar timing with HHS regions in early 2016 and onward. Seasonal influenza B virus patterns in Puerto Rico lagged behind the HHS regions by 2‐10 weeks until the 2016‐2017 season at which point they preceded HHS regions by 0‐4 weeks, showing better synchrony with the HHS regions.

**Figure 2:**
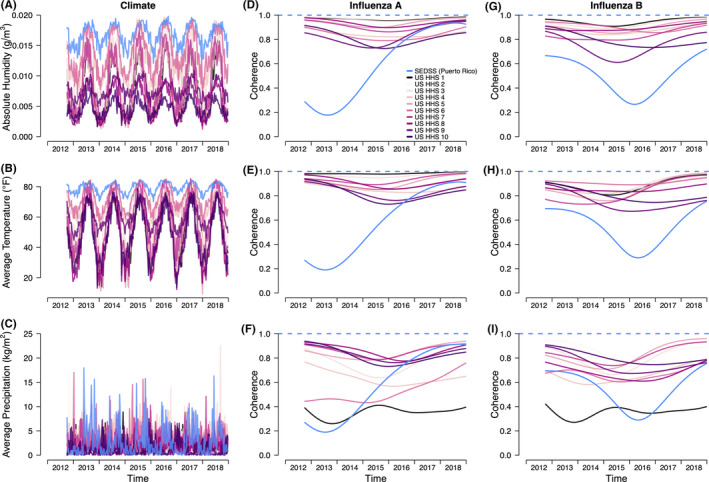
Trends in absolute humidity (A), temperature (B) and precipitation (C) in Puerto Rico and US HHS regions (US WHO/NRVESS) and annual coherence for influenza A (D, E, F) and B (G, H, I). Seasonal decomposition was defined as a 40‐ to 80‐wk period. Colour lines refer to the comparisons of the weekly seasonal time series of SEDSS, Puerto Rico to the weekly seasonal pattern for each climate variable. For coherence, the dashed line at 1 refers to identical seasonal patterns of SEDSS and the climate variables, whereas 0 refers to no association of the two time series

To investigate possible climatic drivers of influenza A and B epidemics patterns within each geographic region, we assessed the coherence and phase differences between location‐specific climate variables and type‐specific influenza incidence (Figure 2A‐I). In terms of the overall time series climatic trends, Puerto Rico was consistently warmer and more humid over the time series compared with weekly average absolute humidity and temperature in HHS regions (Figure 2A‐B). In contrast, precipitation varied greatly across all regions. Some HHS regions had peak rainfall in distinctly different seasons compared with Puerto Rico (Figure 2C). Over the 40‐ to 80‐week component of the time series, we observed high coherence (range: 0.61‐0.99) between absolute humidity and influenza A and B viruses within the HHS regions (Figure 2D, G). However, this pattern was markedly different in Puerto Rico. Influenza A virus coherence was much lower in the early years, but by the 2017‐2018 season, coherence increased to similar levels as the HHS regions (range: 0.18‐0.94). Meanwhile, influenza B virus coherence was higher early in the time series, decreasing in 2015‐2016, and increasing in late 2016, but never having the higher coherence estimates observed in the HHS regions (range: 0.27‐0.72). Similar patterns were observed for coherence between mean weekly temperature and influenza A and B viruses (Figure 2E, H). Coherence between precipitation and influenza varied substantially for the HHS regions and Puerto Rico, but did not have consistently high coherence for the SEDSS Puerto Rico data (Figure 2F‐I). The phase difference analyses showed a high degree of asynchrony of influenza patterns in Puerto Rico and patterns for climate variables (Figure S3C‐D). The seasonal precipitation patterns lagged behind influenza A virus trends in SEDSS by 20 weeks, while absolute humidity and temperature patterns led by about 25‐30 weeks (Figure S3C). For influenza B virus, precipitation, absolute humidity and temperature seasonal components were ahead of the influenza B patterns by 15‐30 weeks (Figure S3D). The seasonal wavelet decomposition for each HHS region showed that influenza peaked when temperature and absolute humidity were low (Figure S4A‐E and K‐O for influenza A and Figure S5A‐E, K‐O for influenza B). For each HHS region, influenza patterns consistently lagged approximately 30 weeks for influenza A and 20 weeks influenza B compared with temperature and absolute humidity patterns (Figure S4F‐J, P‐T, S5F‐J, P‐T).

## 4 DISCUSSION

This is the first study comparing seasonal influenza trends in Puerto Rico to trends in the US HHS regions, and we found influenza trends between HHS regions and SEDSS, Puerto Rico varied over time but increased in synchrony in recent years. We observed distinct synchronization patterns by influenza virus types, showing different dynamics for each type over the time series. For influenza A, the predominant type in Puerto Rico and the HHS regions, the seasonal incidence pattern in Puerto Rico greatly differed from the HHS regions in 2012‐2014, but synchronized beginning in 2015‐2016. Influenza B cases in Puerto Rico had more consistent seasonality and also exhibited increased synchrony starting in the 2016‐2017 season. Relative to influenza in the HHS regions, both virus types in Puerto Rico switched from irregular, asynchronous seasonal epidemics, generally following those in the rest of the United States, to synchronous epidemics, preceding the seasonal epidemics in the continental United States.

One explanation of the seasonal differences between Puerto Rico and the continental United States may be the geographic differences in climate. Consistent with other studies, we found that influenza incidence in HHS regions had high coherence with humidity and temperature. In contrast, Puerto Rico lacked the high coherence with climate observed among the HHS regions, and only had higher coherence during the most recent influenza seasons. The absence of high coherence between influenza peaks and temperature and humidity in Puerto Rico may be because higher year‐round humidity and temperatures have less variation than in temperate regions. In vitro and animal model studies of influenza transmission showed low absolute humidity favours virus survival and aerosolized transmission^19^, ^20^ and epidemiological studies showed lower humidity is associated with the onset of influenza epidemics in the United States.^21^ Increased proximity between susceptible and infected hosts associated with cold weather is also frequently suggested as an important driver of influenza seasonality.^8^ Seasonal variation in host immunity may also explain the seasonality of influenza in temperate countries. Studies also suggest an association between increased influenza activity and the rainy season in several tropical populations,^22^ but seasonal precipitation also had low coherence with influenza activity in Puerto Rico. While climatic conditions may likely affect influenza transmission in some locations, humidity, temperature and precipitation are not the main drivers of influenza in Puerto Rico. Other climatic factors not examined here, or other socio‐demographic factors may be more important drivers, though this needs further examination.

Perhaps, the most striking result of our study was the increased synchronization with HHS regions during recent years for both influenza virus types. As described above, the climate analysis suggests this synchrony was not driven by climate alone and likely influenced by other factors^23^ including travel and global and US influenza viral introductions into Puerto Rico. For instance, a previous study suggested that human mobility has synchronized epidemics among highly connected populations in the United States, with influenza viruses spreading from populous to less populous locations.^17^ It is possible that contact rates at these larger scales may be particularly important to the seasonal patterns of influenza in smaller populations, or for locations that are characterized by less climatic variability, like Puerto Rico. In such cases, seasonal variability in the volume of infected individuals entering a population (like migration from the United States to Puerto Rico during the winter) may exceed any climate‐mediated seasonality.^24^ Analysis of air passenger data and seasonal patterns may help investigate this hypothesis. The time lag of several weeks relative to HHS regions suggests that recent epidemics in Puerto Rico may lead rather than follow those in HHS regions; however, the different geographical scale may complicate this relationship. The SEDSS catchment area is just one region of Puerto Rico, and much smaller than any HHS region. Recent work found that the shape of epidemics varies across different geographies, such that even introductions from another location may result in epidemics with earlier sharper peaks than the source location.^25^ Other studies found highly synchronized epidemics in Australia coincided with years of emergent antigenically distinct subtypes, and synchrony across the continent was a function of global introductions of influenza viruses paired with domestic connectivity.^26^ Our study found that coherence was higher for certain HHS regions compared with others. The high coherence associated with US HHS Region 9 may be due to the fact that Region 9 is comprised of southwestern states (Arizona, California, Nevada), but also Pacific Island states (Hawaii) and territories (American Samoa, Commonwealth of the Northern Mariana Islands, Federated States of Micronesia, Guam, Marshall Islands, and Republic of Palau), and these Pacific Island areas may have similar patterns to Puerto Rico. Similar coherence patterns are also observed when looking at influenza A and B. This may be because even though southern states have warmer and more humid temperatures, they still experience seasonal fluctuations in temperature and humidity that are closer to temperate states compared with tropical locations such as Puerto Rico, and other tropical areas, such as Hawaii or American Samoa.

The annual coherence of influenza B increased from 2015 to the end of 2018. This increase was observed across all comparisons of US HHS Regions and SEDSS in Puerto Rico. Though we did not have data on influenza B lineages, there is evidence from other genomic analyses that the influenza B Victoria lineage had experienced high genetic reassortment in 2016,^27^ which may in part explain the increase in coherence and phase of the influenza B strains across the region. Increases in B Victoria (as opposed to B Yamagata) can also be seen over time in CDC’s virological surveillance from the 2016 to 2017 seasons onward.^28^

This investigation leveraged a large study with consistent recruiting and diagnostic testing over multiple influenza seasons, yet still is subject to several limitations. First, the patient population was limited to two sentinel hospitals and one outpatient clinic in southern Puerto Rico. Because healthcare‐seeking behaviours may differ between populations served by different hospitals, the observed percentages of patients with a respiratory virus may not be representative of all Puerto Rican health facilities. Second, the 6.5 seasons captured through SEDSS are only a snapshot of a long history of influenza in Puerto Rico. For example, it is unclear if the seasons without a regular peak seen, such as those in the earlier years of the study, were common prior to 2012 or if earlier epidemics were more consistent with HHS regions. It is possible that there are cycles working on larger time scales that contribute to sporadic asynchrony or sporadic synchrony. We also could not account for vaccine effects. While Puerto Rico generally has lower influenza vaccination rates compared with US states,^29^ we did not capture vaccination information as part of the surveillance systems and therefore, could not assess its impact the local dynamics. Data on influenza A virus subtypes or B virus lineages were not available for this analysis. We did not examine travel between Puerto Rico and the mainland United States or other locations, but are components that may contribute substantially to influenza dynamics in Puerto Rico. Future studies could include phylogenetic analysis to confirm the timing of influenza strains circulating in the continental United States compared with Puerto Rico. HHS region two includes data from Puerto Rico, which may result in over‐estimating associations with that region; however, the patterns observed for this region were similar to others. We did not have data on influenza trends for all of Puerto Rico and were unable determine if trends in Ponce are similar to the island wide trends. Finally, few participants were 65 years of age and older, and possibly under‐estimating the number of infections in Puerto Rico.^29^

Our study highlights the poorly understood complexity of influenza in tropical regions where climate is not a strong driver of seasonal dynamics. It also broadens our understanding of the relationship between influenza trends in the continental United States and Puerto Rico, and the role of climate. Currently in the United States, influenza vaccines are recommended to be received before the end of October. These recommendations would not have been ideal for Puerto Rico in the early study years. Influenza trends in Puerto Rico will need to be continuously monitored to better understand if these dynamics change again, and to ensure that interventions, like vaccinations, are implemented appropriately for influenza in Puerto Rico.

## CONFLICT OF INTEREST

The authors report no conflict of interest.

## AUTHOR CONTRIBUTIONS

GPB designed the study, contributed to the analyses, participated in the interpretation of results and wrote the first draft of the paper. TQ conducted the wavelet decomposition and phase difference analyses and co‐wrote the paper. LA conducted the descriptive analyses and contributed to the final draft of the paper. SO, LB and ML helped interpret results and edited the final draft of the paper. JM supervised the laboratory testing and reviewed the final draft of the paper. LIA is the principal investigator for the enhanced surveillance system, helped interpret study results and reviewed the final draft of the paper. MJ oversaw the analyses strategy, helped interpret study results and edited the final draft of the paper. All authors revised the draft paper critically for important intellectual content, provided final approval of the version to be published and are accountable for all aspects of the work with regards to accuracy and integrity.

## DISCLAIMER

The findings and conclusions in this report are those of the authors and do not necessarily represent the official position of the Centers for Disease Control and Prevention.

## Supporting information

FigS1‐S5

Table S1
