# Supplementary material for: Recent influenza activity in tropical Puerto Rico has become synchronized with mainland US
Source: Influenza Other Respir Viruses. 2020 Jul 2;14(5):515–23. doi: 10.1111/irv.12744 (PMC7431640; doi:10.1111/irv.12744)
Supplement: Supplementary file 1 — FigS1‐S5 [file IRV-14-515-s001.docx]

**Supplementary Information**

Supplemental Figure 1. Map of United States Department of Health and Human Services regions and location of SEDSS Site in Ponce, Puerto Rico.

Supplemental Figure 2. Seasonal wavelet decomposition of each: influenza all types (a), influenza A (b), and influenza b (c) in Puerto Rico and US HHS regions (U.S. WHO/NRVESS). Seasonal decomposition was defined as a 40-56 week period. Color lines refer to the seasonal pattern extracted for each region.

Supplemental Figure 3. Seasonal wavelet decomposition for absolute humidity, temperature, and precipitation and influenza A (A) and B (B) in Puerto Rico, and phase differences between climate variables and influenza A (C) and B (D). Seasonal decomposition was defined as 40-80 week period. Colors refer to the comparison of seasonal time series between SEDSS influenza patterns and each of the climate variables, where influenza cases are leading the climate patterns if positive, and lagging behind if negative.

Supplemental Figure 4. Seasonal trends for absolute humidity, temperature, and precipitation and influenza A within each US HHS regions (U.S. WHO/NRVESS) (a-e and k-o), and phase differences between climate variables and influenza A within each HHS region (f-j and p-t). Seasonal decomposition was defined as 40-80 week period. Colors refer to the comparison of seasonal time series between influenza patterns and each of the climate variables within each HHS region, where influenza cases are leading the climate patterns if positive, and lagging behind if negative.

Supplemental Figure 5. Seasonal trends for absolute humidity, temperature, and precipitation and influenza B within each US HHS regions (U.S. WHO/NRVESS) (a-e and k-o), and phase differences between climate variables and influenza B within each HHS region (f-j and p-t). Seasonal decomposition was defined as 40-80 week period. Colors refer to the comparison of seasonal time series between influenza patterns and each of the climate variables within each HHS region, where influenza cases are leading the climate patterns if positive, and lagging behind if negative.
