# Supplementary material for: Recent influenza activity in tropical Puerto Rico has become synchronized with mainland US
Source: Influenza Other Respir Viruses. 2020 Jul 2;14(5):515–23. doi: 10.1111/irv.12744 (PMC7431640; doi:10.1111/irv.12744)
Supplement: Supplementary file 2 — Table S1 [file IRV-14-515-s002.docx]

**Supplemental Table 1. Average annual coherence of influenza, and types A and B between SEDSS in southern Puerto and US HHS Regions from May 2012 to Dec 2018.**

| **US HHS Regions** | **Average annual coherence with influenza*** | **Average annual coherence with influenza A*** | **Average annual coherence with influenza B*** |
| --- | --- | --- | --- |
| Region 1 | 0.56 | 0.71 | 0.72 |
| Region 2 | 0.66 | 0.73 | 0.76 |
| Region 3 | 0.58 | 0.70 | 0.71 |
| Region 4 | 0.52 | 0.69 | 0.67 |
| Region 5 | 0.57 | 0.70 | 0.70 |
| Region 6 | 0.56 | 0.66 | 0.74 |
| Region 7 | 0.58 | 0.69 | 0.68 |
| Region 8 | 0.52 | 0.68 | 0.65 |
| Region 9 | 0.67 | 0.72 | 0.71 |
| Region 10 | 0.60 | 0.69 | 0.68 |

* Reference is SEDSS in southern Puerto Rico
